# Single-cell landscape identified *SERPINB9* as a key player contributing to stemness and metastasis in non-seminomas

**DOI:** 10.1038/s41419-024-07220-5

**Published:** 2024-11-11

**Authors:** Zhouliang Bian, Biying Chen, Guohai Shi, Haihua Yuan, Yue Zhou, Bin Jiang, Long Li, Hengchuan Su, Yanjie Zhang

**Affiliations:** 1 Department of Oncology, Ninth People’s Hospital, Shanghai Jiao Tong University School of Medicine, Shanghai, 201900 PR China; 2 Shanghai Institute of Precision Medicine, Ninth People’s Hospital, Shanghai Jiao Tong University School of Medicine, Shanghai, 200125 PR China; 3 Department of Urology, Fudan University Shanghai Cancer Center, Shanghai, 200032 PR China; 4 Department of Oncology, Shanghai Medical College, Fudan University, Shanghai, 200032 PR China; 5 School of Biomedical Engineering, Shanghai Jiao Tong University, Shanghai, 200030 PR China; 6 Department of Urology, Ninth People’s Hospital, Shanghai Jiao Tong University School of Medicine, Shanghai, 200011 PR China

**Keywords:** Germ cell tumours, Cell biology

## Abstract

Embryonal carcinoma (EC), characterized by a high degree of stemness similar to that of embryonic stem cells, is the most malignant subtype within non-seminomatous testicular germ cell tumors (TGCTs). However, the mechanisms underlying its malignancy remain unknown. In this study, we employed single-cell RNA sequencing to analyze four non-seminoma samples. Our differential expression analysis revealed high expression of *SERPINB9* in metastatic EC cells. We conducted in vitro experiments to further investigate *SERPINB9*’s role in the progression of EC. Functionally, the knockdown of *SERPINB9* in NCCIT and NTERA-2 leads to a diminished migratory capability and decreased cis-platin resistance, as demonstrated by Transwell migration assay and drug sensitivity assay. Moreover, embryoid bodies showed reduced size and lower OCT4 expression, alongside heightened expression of differentiation markers AFP, ACTA2, and CD57 in sh*SERPINB9* cells. In vivo, the role of *SERPINB9* in maintaining cancer stemness was validated by the limiting dilution assay. Mechanistically, Bulk RNA-seq further showed downregulation of ERK1/2 signaling and WNT signaling pathways with concomitant upregulation of differentiation pathways subsequent to *SERPINB9* knockdown. Additionally, the analysis indicated increased levels of cytokines linked to tertiary lymphoid structures (TLS), such as *IL6*, *IL11*, *IL15*, *CCL2*, *CCL5*, and *CXCL13* in sh*SERPINB9* cells, which were further validated by ELISA. Our research indicates that *SERPINB9* plays a key role in driving tumor progression by enhancing tumor stemness and suppressing TLS. This study stands as the first to elucidate the molecular signature of non-seminomas at a single-cell level, presenting a wealth of promising targets with substantial potential for informing the development of future therapeutic interventions.

## Introduction

Testicular germ cell tumors (TGCTs) constitute the predominant cancer affecting young males aged between 15 and 40 years, comprising a significant health concern [1]. TGCTs encompass two primary categories: seminomas and non-seminomas. The non-seminomatous group consists of embryonal carcinoma (EC), teratoma, yolk sac tumor, and trophoblastic tumor. Notably, non-seminomas exhibit a heightened propensity for metastasis when compared to seminomas, with approximately 40% of non-seminoma cases presenting as clinical stage II or III diseases [2]. Consequently, non-seminoma patients generally face a worse prognosis compared to their seminomatous counterparts. Prior studies have shown that advanced non-seminoma patients, particularly those categorized as poor prognosis according to the International Germ Cell Cancer Collaborative Group (IGCCCG) classification, exhibit a 3-year overall survival rate of 72% [3]. Nevertheless, the factors contributing to the worse prognosis of non-seminoma patients have yet to be elucidated.

Notably, the metastatic spread in non-seminomas is predominantly lymphatic, with retroperitoneal lymph nodes serving as a primary site of metastasis. The presence of undetectable metastases in retroperitoneal lymph nodes significantly contributes to non-seminoma relapse and worsened relapse-free survival [4]. Interestingly, previous study found a positive relationship between increased risk of metastases and EC [5]. However, the exact molecular mechanism of the tendency of metastasis in non-seminomas is still unclear.

Among the non-seminomatous subtypes, EC represents the stem cell component and is notably aggressive [6]. A more profound exploration of this specific tumor population may offer valuable insights for the therapeutic management of non-seminomas. However, the high prevalence of mixed histology in non-seminomas poses a significant challenge for traditional research approaches reliant on bulk RNA sequencing [7]. In this context, single-cell RNA sequencing (scRNA-seq) emerges as a promising technique, providing the ability to scrutinize the transcriptome at the level of individual cells.

Furthermore, the pathogenesis and metastasis of TGCTs are substantially influenced by the complexities of their microenvironment [8]. The testis, as an immune-privileged site, inherently shields germ cells from the immune system’s scrutiny [9]. Earlier investigations have elucidated the rich infiltration of immune cells in seminomas and disparities in inflammatory levels between early and advanced seminoma stages [10]. While the involvement of the immune system in non-seminomas remains a relatively uncharted territory.

In this study, we conducted scRNA-seq analysis on four primary non-seminoma tumors collected from patients exhibiting diverse metastatic conditions. Our findings highlighted the elevated expression of *SERPINB9* in metastatic EC cells within non-seminoma patients. Consistently, knockdown of *SERPINB9* in EC cell lines decreased their migration capacity. In addition, drug sensitivity assay showed decreased cis-platin resistance after knockdown of *SERPINB9*. Furthermore, we also showed the pivotal role of *SERPINB9* in stemness maintenance through embryoid body formation assay, limiting dilution assay and RNA-seq analysis. Additionally, our study identified the role of *SERPINB9* in suppression of tertiary lymphoid structure (TLS)-associated cytokines and promoting tumor progression. In sum, our study provided valuable insights for the molecular mechanisms underlying the development of non-seminomas.

## Results

### Analysis of scRNA-seq datasets of primary non-seminomas uncovered heterogeneous cell composition

**Fig. 1:**
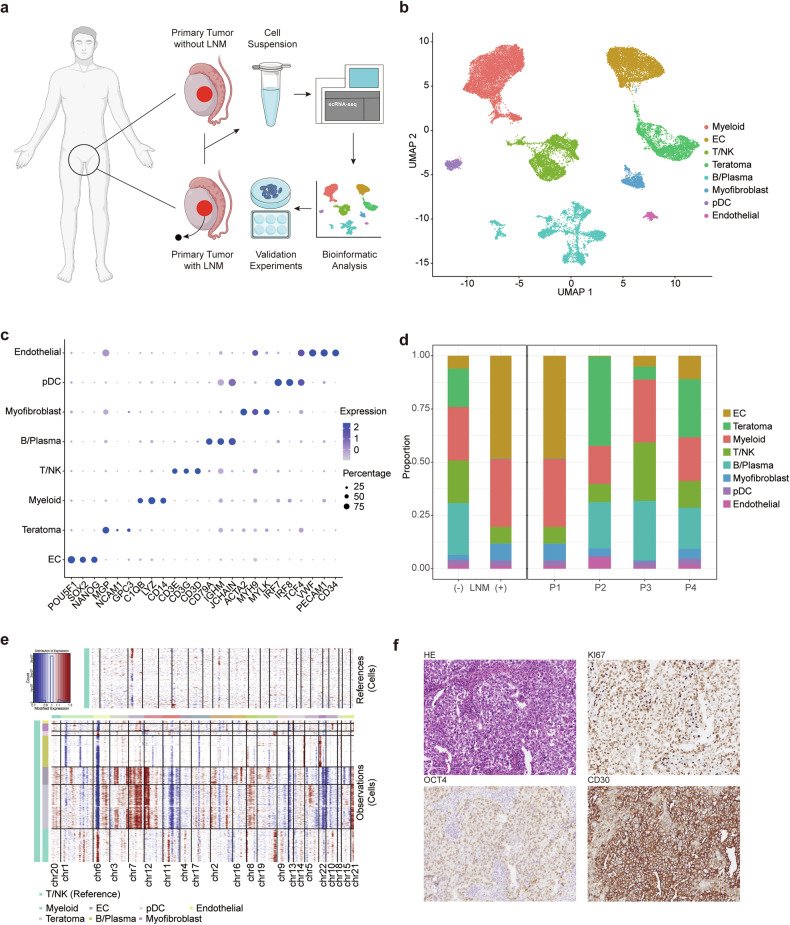
Identification of cellular composition in non-seminomas. **a** Schematic workflow describing the process of sample collection, analysis and validation. **b** UMAP plot of cells from non-seminoma samples colored by cell populations. **c** Dot plot of expression levels of canonical marker genes. **d** Bar plot summarizing the cellular proportion for four non-seminoma samples. **e** Heatmap showing copy number variation in malignant tumor cells from P4. **f** HE and IHC staining showing classical markers including OCT4 and CD30 of EC in P1 (scale bar: 100 µm).

In an effort to elucidate the intricate molecular mechanisms underpinning the metastasis of non-seminoma tumors, we employed single-cell RNA sequencing (scRNA-seq) to analyze four primary non-seminoma samples. These samples comprised one that had metastasized to retroperitoneal lymph nodes (P1) and three that displayed no metastatic behavior (P2-4) (Fig. 1a). A total of 27,358 cells (8502 in metastatic group and 18,856 in non-metastatic group) were kept for further analysis after filtering out low-quality cells. Upon executing principal component analysis (PCA) and leveraging graph-based clustering methodologies, we discerned 8 distinct cell clusters (Fig. 1b). These clusters were corroborated using classical markers (Fig. 1c). Specifically, we identified: EC cells (*POU5F1*, *SOX2* and *NANOG*), teratoma cells (*MGP*, *NCAM1* and *GPC3*), myeloid cells (*C1QB*, *LYZ* and *CD14*), T/NK cells (*CD3E*, *CD3G* and *CD3D*), B/Plasma cells (*CD79A*, *IGHM* and *JCHAIN*), myofibroblasts (*ACTA2*, *MYH9* and *MYLK*), pDC (*IRF7*, *IRF8* and *TCF4*) and endothelial cells (*VWF*, *PECAM1* and *CD34*) (Fig. 1c; Fig. S1). Interestingly, the tumor compartment of P1 consisted exclusively of EC (Fig. 1d). Further, we employed InferCNV to gauge the copy number variations, which provided robust confirmation of the identities of both EC and teratoma cells (Fig. 1e; Fig. S2). The immunohistochemistry (IHC) analysis showed positive expression of EC markers including OCT4 and CD30 in all four patients (Fig. 1f; Fig. S3).

### Deciphering differentiation pathways in non-seminomas

**Fig. 2:**
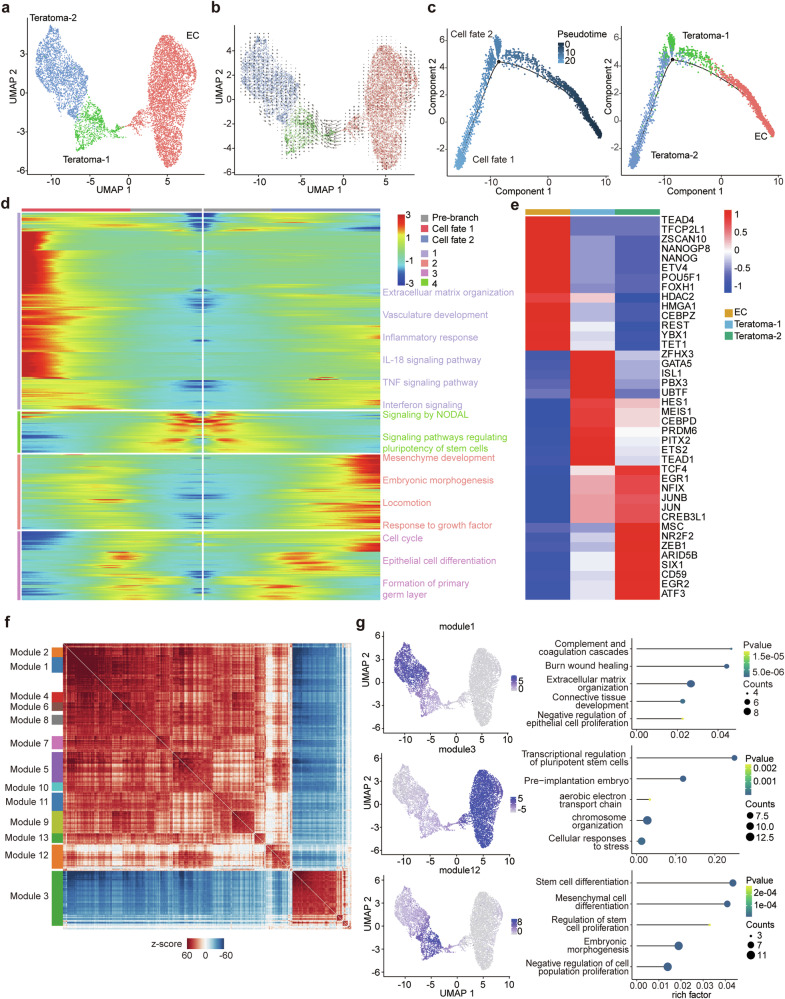
Tumor heterogeneity captured by scRNA-seq. **a** UMAP plot of malignant tumor cells colored by clusters. **b** RNA velocity analysis showing differentiation from EC to teratoma. **c** Monocle pseudotime trajectory of non-seminoma tumor cells. **d** Branched expression heatmap showing significantly changed genes identified by the BEAM function at the branch point. **e** Heatmap of transcription factor regulon activities estimated by SCENIC. **f** Genes with significant autocorrelation were grouped into 13 gene modules. **g** UMAP plots showing the representative gene module activities for three tumor subsets and dot plots showing pathway enrichment of genes in the gene modules.

The role of EC cells as cancer stem cells in non-seminomas has been poorly understood. To better understand the changing expression patterns throughout non-seminoma development, we conducted a thorough analysis of non-seminoma tumor cells. This led to the discovery of three distinct cellular sub-clusters including EC, teratoma-1 and teratoma-2 (Fig. 2a). Leveraging RNA velocity analysis, we elucidated a clear differentiation trajectory initiating from the EC subset and culminating in teratoma-2 (Fig. 2b). Further pseudotime trajectory analysis using Monocle 2 provided a nuanced differentiation landscape of the teratoma subsets (Fig. 2c). Notably, teratoma-2 emerged as the more differentiated subset, whereas teratoma-1 reflected a relatively primitive state in comparison. Delving deeper into the molecular underpinnings, our branched expression analysis spotlighted the activation of key signaling pathways, specifically the TNF and IL-18 pathways, in the more differentiated teratoma-2 (Fig. 2d). Conversely, the trajectory towards teratoma-1 was hallmarked by the enrichment of pathways implicated in embryonic morphogenesis, growth factor responsiveness, and primary germ layer formation. Intriguingly, teratoma-1 showcased an expression signature reminiscent of immature teratomas, underscored by its expression of *GPC3* - a marker previously associated with immature teratoma [11]. Utilizing the SCENIC analysis on the non-seminoma tumor cells, we observed heightened activity of transcription factors quintessential for pluripotency maintenance, notably *NANOG*, *POU5F1*, and *HDAC2*, within the EC subset (Fig. 2e) [12]. Teratoma-1 was characterized by a pronounced activity of endodermal and mesodermal transcription factor, inclusive of *GATA5*, *ISL1*, and *PITX2* [13–15]. *PBX3* and *MEIS1* were discernibly activated in teratoma-1, echoing findings from previous study that highlighted their synergistic oncogenic potential [16]. Accordingly, gene modules associated with pluripotent stem cells were activated in EC subset while differentiation-associated gene modules were identified in teratoma subsets (Fig. 2f, g). In sum, our study identified critical factors that contribute to the preservation and differentiation of EC cells. These cells constitute the stem cell population linked to metastasis and poorer prognoses. These findings provided novel therapeutic targets in the treatment of non-seminomas.

### *SERPINB9* was associated with metastatic phenotype and stemness of EC

**Fig. 3:**
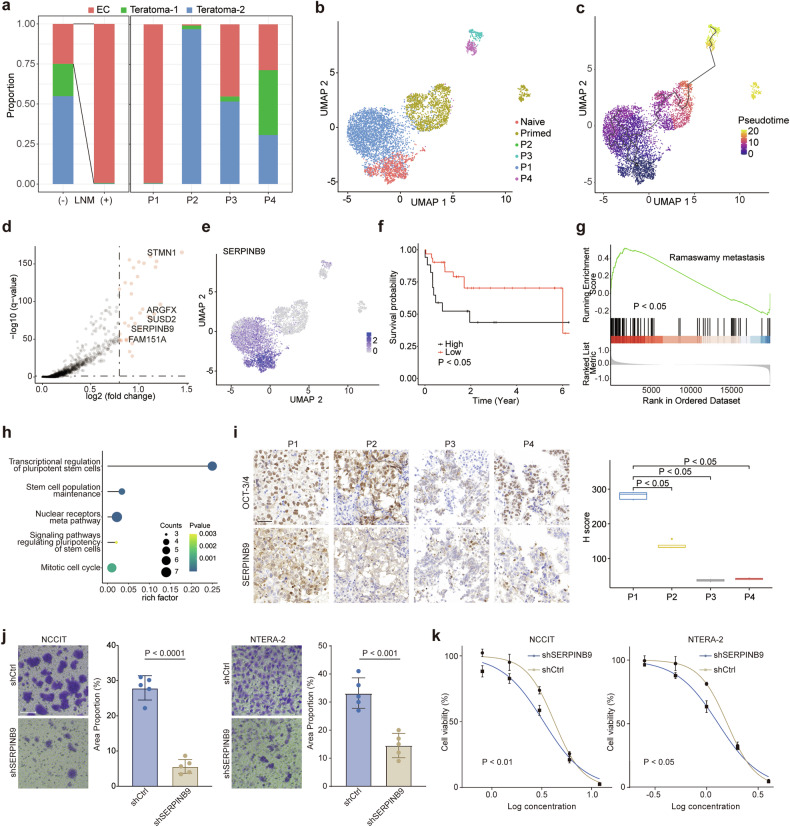
*SERPINB9* was identified to be associated with stemness in scRNA-seq dataset. **a** Bar plot showing the cellular proportion of non-seminoma tumor cells in four samples. **b** UMAP plot of EC and hESC cells colored by cell types. **c** UMAP plot colored by pseudotime inferred by Monocle 3 showing the differentiation trajectory from hESC to EC. **d** Volcano plot showing higher expression level of *SERPINB9*, *SUSD2* and *ARFX* in EC cells of primary tumor from metastatic patient. **e** Featureplot showing the expression pattern of *SERPINB9* in hESC and EC cells. **f** Survival curves showing *SERPINB9* expression associated with worse DFI in TCGA TGCT non-seminoma patients (n(high) = 17, n(low) = 31; *P* value: Gehan-Breslow-Wilcoxon test). **g** GSEA analysis showed enrichment of metastasis-associated pathway in samples with higher *SERPINB9* expression from TCGA non-seminoma dataset. **h** Dot plot showing up-regulation of pathways associated with pluripotent stem cells in samples with higher *SERPINB9* expression from TCGA non-seminoma dataset. **i** IHC staining showing stronger staining of SERPINB9 on EC cells expressing OCT4 in primary tumor from metastatic patient. Boxplot showing higher H-score of SERPINB9 for sample with metastasis (scale bar: 50 µm; *P* value: Wilcoxon test with Bonferroni’s correction). **j** Transwell migration assay showing decrease of migratory capability of NCCIT and NTERA-2 after knockdown of *SERPINB9* (scale bar: 50 µm; *P* value: Welch’s *t*-test). **k** Drug sensitivity assay showing decrease of IC50 after the knockdown of *SERPINB9* (IC50 for NCCIT: 4.213 µM vs 3.295 µM, IC50 for NTERA-2: 2.386 µM vs 1.950 µM; *P* value: Welch’s *t*-test).

To elucidate the molecular mechanisms of non-seminoma metastasis, we comprehensively evaluated the tumor cell composition across variable metastatic conditions (Fig. 3a). Intriguingly, pure EC typified the metastatic non-seminoma profile, whereas the non-metastatic counterpart was dominated by the more differentiated teratoma components. This observation prompted the postulation that EC cells with a heightened metastatic propensity might exhibit an amplified stemness signature. Reinforcing this hypothesis, integrated analyses with human embryonic stem cells (hESC) revealed a resemblance of metastatic EC cells to naïve hESCs (Fig. 3b). Pseudotime trajectory analytics further corroborated this, indicating a pronounced differentiation gradient in EC cells from conditions P2-4 compared with those from P1 (Fig. 3c). Differential expression analysis spotlighted an enriched expression of naïve hESC markers such as *SUSD2* and *ARGFX* in metastatic EC cells (Fig. 3d) [17, 18]. An additional layer of complexity was unveiled with the notable overexpression of *SERPINB9*, a previously documented modulator of cancer stem cell pluripotency, in metastatic EC (Fig. 3e) [19]. Interestingly, public ATAC-seq datasets showed more open state in *SERPINB9* promoter region in embryonic stem cell lines compared to those in more differentiated cells (Fig. S4a). Survival analysis using non-seminoma patients from TCGA TGCT dataset showed the relationship between *SERPINB9* expression and worse disease-free interval (DFI) (Fig. 3f). In addition, the expression of *SERPINB9* was higher in stage II-III patients compared to stage I patients (Fig. S4b). Pathway enrichment analyses further revealed enrichment of pathways associated with metastasis and the transcriptional regulation of pluripotent stem cells in samples with higher *SERPINB9* expression, reinforcing the findings from scRNA-seq dataset analyses (Fig. 3g, h).

To robustly validate the SERPINB9 expression at the protein level, we embarked on IHC analyses of primary tumor samples from the four patients (Fig. 3i). IHC slides of the metastatic patient showed strongest SERPINB9 staining, suggesting its role in stemness maintenance and metastasis. To elucidate the function of *SERPINB9* in metastasis, Transwell migration assay was performed (Fig. 3j; Fig. S4c–f). The migratory ability of two EC cell lines, NCCIT and NTERA-2 was attenuated post-*SERPINB9* knockdown (Fig. 3j; Fig. S4c–f). Building on previous research that highlighted the link between cancer stem cells and drug resistance, we examined the role of *SERPINB9* in chemoresistance. Drug sensitivity assays utilizing cisplatin, commonly prescribed in non-seminoma treatments, were conducted. (Fig. 3k; Fig. S4g,h). The IC50 for NCCIT and NTERA-2 decreased significantly after the knock down of *SERPINB9*. Additionally, the cell proliferation assay demonstrated significantly reduced growth of NCCIT and NTERA-2 cells following *SERPINB9* knockdown, further supporting the role of *SERPINB9* in promoting tumor growth (Fig. S4i–n).

**Fig. 4:**
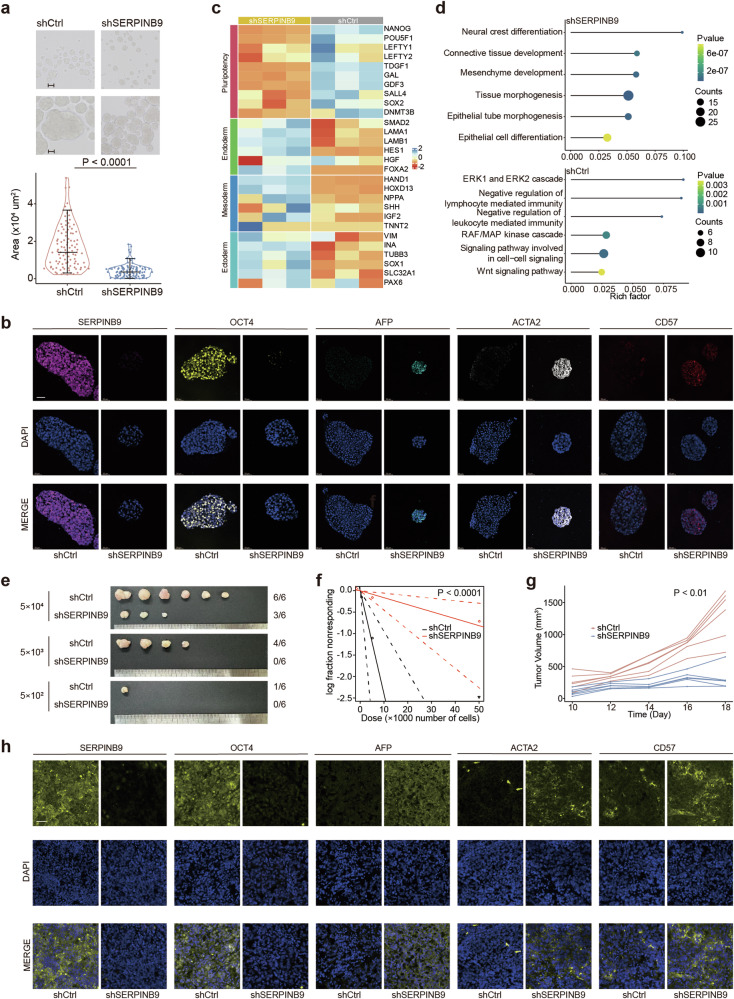
Role of *SERPINB9* in supporting stem cell maintenance as evidenced by in vitro and in vivo studies. **a** Representative images for embryoid bodies from shCtrl and sh*SERPINB9* NCCIT cells (scale bar: 100 µm (upper panel), 50 µm (lower panel)). Bar plot showing the area of embryoid bodies (*P* value: Wilcoxon rank-sum test). **b** IF of embryoid bodies from shCtrl and sh*SERPINB9* NCCIT cells (scale bar: 50 µm). **c** Heatmap showing the up-regulation of differentiation-associated genes and down-regulation of pluripotency-associated genes after the knockdown of *SERPINB9*. **d** Dot plot showing up- and down-regulated signaling pathway after *SERPINB9* knockdown. **e** Representative images of tumors from the limiting dilution assay. **f** CSC frequency analysis using ELDA software (1/stem cell frequency (95%CI): shCtrl: 4,158 (10,571 - 1,635); sh*SERPINB9*: 74,214 (233,999 - 23,538)). **g** Tumor growth curves using tumor volumes from each group of the CDX models (*P* value: Wilcoxon test). **h** Representative images of F-IHC staining for SERPINB9, OCT4, AFP, ACTA2 and CD57 for the CDX tumor tissues (scale bar: 50 µm).

To further validate the role of *SERPINB9* in stemness maintenance, embryoid formation assay was performed using NCCIT. Decreased size and number of embryoid bodies were observed after the knockdown of *SERPINB9* (Fig. 4a; Fig. S5a). The immunofluorescence (IF) of embryoid bodies showed weaker staining of stemness-associated marker (OCT4) and stronger staining of differentiation-associated markers (AFP, ACTA2 and CD57) (Fig. 4b; Fig. S5b–f). Further investigation of embryoid bodies using bulk RNA-seq revealed an attenuated expression of quintessential pluripotency-associated genes such as *NANOG*, *POU5F1*, and *SALL4* after *SERPINB9* knockdown (Fig. 4c). In parallel, genes associated with tri-germ layer differentiation, spanning endoderm, mesoderm and ectoderm, were upregulated in sh*SERPINB9* group. Pathway enrichment analysis spotlighted the activation of molecular cascades pivotal for neural crest differentiation, mesenchymal evolution, and epithelial differentiation post-*SERPINB9* knockdown (Fig. 4d). In addition, *SERPINB9* knockdown led to the downregulation of immunosuppressive pathways. While ERK1/2 cascade and WNT signaling pathway were enriched in shCtrl group. The analyses of RNA-seq datasets and qPCR showed that genes related to ERK1/2 signaling and WNT signaling pathways were downregulated after *SERPINB9* knockdown (Fig. S5g–j). The investigation of TCGA non-seminoma datasets showed enrichment of ERK1/2 cascade and canonical WNT signaling pathways in tumor samples with higher expression of *SERPINB9* (Fig. S5k, l). Further IF analyses showed decreased expression of p-ERK and β-catenin in embryoid bodies after *SERPINB9* knockdown (Fig. S5m, n). To strengthen the evidence for SERPINB9’s role in maintaining cancer stemness, a limiting dilution assay, the gold standard for assessing the self-renewal capacity of cancer stem cells (CSCs) in vivo, was performed (Fig. 4e, f; Fig. S6a–c). Tumor incidence was significantly reduced across all three tested dilutions in the sh*SERPINB9* group compared to shCtrl group (Fig. 4e). Moreover, the frequency of CSCs calculated by Extreme Limiting Dilution Analysis (ELDA) was notably lower in the sh*SERPINB9* group, further supporting the involvement of *SERPINB9* in promoting stemness (Fig. 4f).

To further investigate the role of *SERPINB9* in tumor growth and proliferation in vivo, a cell line-derived xenograft (CDX) was established using NCCIT cells with shCtrl or sh*SERPINB9* (Fig. 4g; Fig. S6d, e). Upon *SERPINB9* knockdown, the NCCIT cells exhibited a marked reduction in tumor growth. Comparative analyses revealed that the xenograft tumors derived from sh*SERPINB9* NCCIT cells were significantly lighter and smaller in volume than shCtrl cells (Fig. 4g; Fig. S6d). Moreover, fluorescent IHC (F-IHC) analyses revealed a diminished intensity of staining for the stemness-related marker OCT4, alongside an intensified staining for markers indicative of differentiation (AFP, ACTA2 and CD57) in xenograft tumor tissues following *SERPINB9* knockdown (Fig. 4h; Fig. S6f–j). Furthermore, F-IHC also showed decreased expression of p-ERK and β-catenin after *SERPINB9* knockdown (Fig. S6k, l). These results indicated the cardinal role of *SERPINB9* in metastasis and maintenance of stemness in EC.

### Decoding the cellular complexity of non-seminoma

**Fig. 5:**
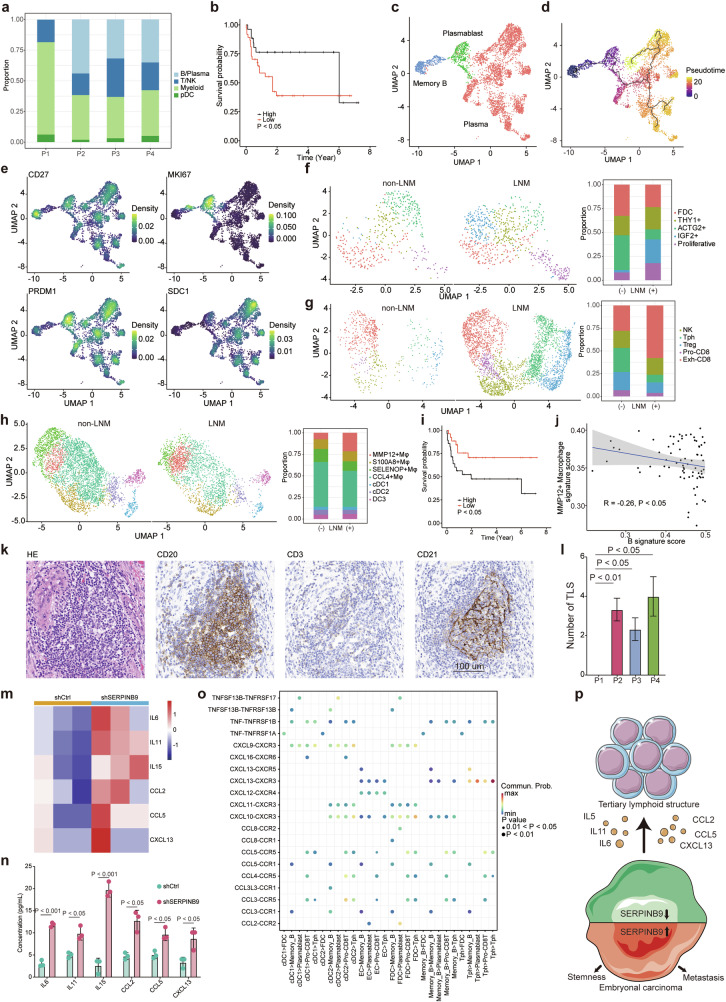
Immune and mesenchymal cell subsets presented in the tumor microenvironment of non-seminomas. **a** Bar plot summarizing the cellular proportion of immune cells in non-seminomas. **b** Survival plot showing association of B/Plasma-associated gene signature with better PFI in TCGA TGCT non-seminoma patients (n(high) = 28, n(low) = 26; *P* value: Gehan-Breslow-Wilcoxon test). **c** UMAP plot showing the cell sub-populations in B/Plasma cells. **d** UMAP plot colored by pseudotime estimated by Monocle 3 showing the differentiation trajectory from memory B cell to plasma cells. **e** UMAP plot showing expression levels of key marker genes of B/Plasma cells. **f** UMAP plot of mesenchymal cells colored by sub-clusters. Bar plot summarizing the proportion of mesenchymal cell subsets. **g** UMAP plot of NK/T cells colored by sub-clusters. Bar plot summarizing the proportion of NK/T cell subsets. **h** UMAP plot of myeloid cells colored by sub-clusters. Bar plot summarizing the proportion of myeloid cell subsets. **i** Survival curves plot showing association of *MMP12*+ macrophage-associated gene signature with worse PFI (n(high) = 22, n(low) = 32; *P* value: Gehan-Breslow-Wilcoxon test). **j** Scatter plot showing inverse correlation between samples scored by gene signatures derived from *IGF2*+ myofibroblasts and B cells in TCGA TGCT non-seminoma dataset using Spearman’s correlation (*P* value and correlation coefficient: Spearman’s correlation test). **k** HE and IHC staining showing presence of TLS indicated by CD20 + B cells, CD3 + T cells and CD21 + FDC in primary tumors from non-metastatic patients. **l** The number of TLS structures in non-seminoma samples (*P* value: Welch’s *t*-test). **m** Up-regulation of cytokines after knockdown of *SERPINB9* in RNA-seq dataset. **n** Up-regulation of cytokines after knockdown of *SERPINB9* using ELISA (*P* value: Welch’s *t*-test). **o** Cell-cell communication among cell clusters in non-metastatic non-seminomas. **p** Schematic illustration of *SERPINB9*’s role in progression of non-seminomas.

The intricate cellular landscape of the TGCT microenvironment has remained enigmatic. While earlier works alluded to variable degrees of B-cell infiltration in TGCTs, our analysis offered a more detailed insight [20]. Specifically, in non-metastatic non-seminomas, B/Plasma cells were not only evident but were represented in proportions akin to myeloid cells (Fig. 5a). Contrasting this, metastatic sample exhibited a conspicuous absence of B/Plasma cells. Corroborating this observation, patients demonstrating elevated signatures of B/Plasma cell expression consistently registered enhanced progression-free intervals (PFI) (Fig. 5b). Given the intrinsic association of B cells with TLS within tumor milieus, we embarked on a meticulous sub-clustering of B/Plasma cells [21]. This stratification unraveled B cells spanning the entire maturation continuum, encompassing memory B cells, plasmablasts, and plasma cells, in non-metastatic non-seminomas (Fig. 5c, d). The identity of these B/Plasma cell subsets was ascertained utilizing classical markers: *CD27* for memory B cells, *MKI67* and *PRDM1* for plasmablasts, and *SDC1* for plasma cells (Fig. 5e). It is worth noting that antibodies secreted by plasma cells are posited to modulate anti-tumorigenic immune responses [22]. Extending our inquiry to other TLS constituents, we observed a pronounced presence of follicular dendritic cells (FDC) and peripheral helper cells (Tph) in non-metastatic specimens (Fig. 5f, g; Fig. S7a, b). Notably, a distinct cluster of *IGF2*+ myofibroblasts was exclusive to the metastatic cohort, hinting at a potential association with tumor progression (Fig. 5f). Beyond the realm of TLS, myeloid cells, recognized orchestrators of tumor development, merited attention. In this study, myeloid cells were more abundant in metastatic non-seminoma (Fig. 5a). A further sub-clustering elucidated various myeloid subsets, notably highlighting an elevated representation of *MMP12*+ macrophages in metastatic sample (Fig. 5h; Fig. S7c). The gene signature associated with *MMP12*+ macrophages was associated with worse PFI in TCGA TGCT non-seminoma patients (Fig. 5i). *MMP12* expression induced the EMT reprogramming and contributed to metastasis in lung cancer [23]. However, its precise role within myeloid cells remains ambiguous. In order to further elucidate the relationships among distinct cellular constituents within non-seminomas, we used gene signatures associated with *MMP12*+ macrophage, *IGF2*+ myofibroblast and B cell to score non-seminoma samples from TCGA TGCT dataset (Fig. 5j; Fig. S8a). The analysis unveiled a marked inverse correlation between the gene signature scores of *IGF2*+ myofibroblasts/*MMP12*+ macrophages and those of B cells, suggesting their possible relationship. To validate the role of TLS in the metastasis of non-seminomas, IHC was performed in the four non-seminoma patients. Organized TLS structures, evidenced by B cells (CD20), T cells (CD3), and FDC (CD21) markers, were prominent in non-metastatic samples (Fig. 5k, l). While the expression of CD3, CD21 and CD20 was low in metastatic sample (Fig. S8b). To identify the role of *SERPINB9* in EC for orchestrating an immunosuppressive milieu, RNA-seq dataset of *SERPINB9* knockdown and control NCCIT was examined. The expression levels of *IL6*, *IL11*, *IL15*, *CCL2*, *CCL5* and *CXCL13*, which are important cytokines for development of B cells and TLS, were higher after the knockdown of *SERPINB9* (Fig. 5m) [24–29]. The expression levels of these cytokines were significantly increased after the knockdown of *SERPINB9* using enzyme-linked immunosorbent assay (ELISA) (Fig. 5n). These findings suggested that *SERPINB9* in EC may be important in inhibition of anti-neoplastic TLS.

In order to identify the cellular interactions linked to metastasis in non-seminomas, a detailed analysis of cell-cell communications was conducted (Fig. S8c). EC cells appeared to preserve their inherent stemness via the WNT5B-FZD3/5 interaction, as suggested by prior research [30]. *IGF2*+ myofibroblasts might also contributed to the stemness of EC cells through WNT6 signaling. Intriguingly, a heightened proportion of *IGF2*+ myofibroblasts was discernible in metastatic non-seminomas, corroborating their comparably undifferentiated phenotype. These myofibroblasts seemed to orchestrate the chemoattraction of *MMP12*+ macrophages into the tumor milieu via the CCL8-CCR1 signaling axis [31]. Subsequent recruitment of Treg cells by these macrophages is facilitated through CCL3/4-CCR5 and CXCL9-CXCR3 pathways, with subsequent Treg activation mediated by CD80-CTLA4/CD28 interactions [32–34]. On the other hand, in non-metastatic non-seminomas, both EC and immune cells participated in the formulation of TLS through multifaceted communications (Fig. 5o, p). Consistent with previous studies, key molecule *CXCL13*, primarily secreted by Tph, recruited memory B cells and plasmablasts into the TLS [35]. Interestingly, non-metastatic EC cells also expressed *CXCL13* and *CCL5* for recruiting B cells. Moreover, Tph were implicated in fostering FDC development via TNF-TNFRSF1A interactions, and they utilized the CXCL13-CXCR3 and CCL3/4/5-CCR5 axes to recruit proliferative CD8 T cells, thus augmenting anti-tumoral responses [36, 37]. In the tumor microenvironment of non-metastatic non-seminomas, *TNFSF13B* was highly expressed on FDC, cDC1 and cDC2 and promoted the proliferation through the receptors *TNFRSF13B* and *TNFRSF17* [38]. Molecules such as *CXCL9/10/11*, released by cDC2 and FDC, have demonstrated roles in the migration, differentiation, and activation of anti-neoplastic immune cells like B cells and proliferative CD8 T cells [39]. The significant correlation between TLS presence and favorable prognosis in non-seminomas propounds the therapeutic induction of TLS as a potential avenue to curb tumor progression and enhance patient outcomes. Experiments on murine models have shown the feasibility of local TLS induction using associated molecules like *CXCL13* [40]. Collectively, these results suggested a transition from a TLS-associated anti-neoplastic milieu to an immunosuppressive environment. Notably, this change was associated with increased levels of *SERPINB9* in EC cells.

## Discussion

TGCTs represent a distinct subset of malignancies predominantly afflicting the young male demographic. Among the two main categories of TGCTs, non-seminomas are of particular concern, due to their comparatively unfavorable prognosis and augmented metastatic inclination. Notwithstanding the clinical significance, the transcriptional landscape of non-seminomas remains largely uncharted at single-cell resolution primarily attributable to the rarity of these tumors. We harnessed the power of scRNA-seq to delineate the critical role of *SERPINB9* in modulating the metastatic potential and the maintenance of stemness in EC cells.

Despite being the most malignant component in non-seminomas, prior research on EC has been limited by the challenges of discerning the complex composition of tumor cells using bulk RNA-seq. In this study, we harnessed the high resolution of scRNA-seq to directly identify EC and investigate their molecular characteristics. This approach led us to discover the pivotal role of *SERPINB9* in non-seminoma metastasis and stemness maintenance. *SERPINB9* belongs to the family of serine protease inhibitors, implicated in diverse physiological processes [19]. Previous research indicates that *SERPINB9* is markedly expressed in tertiary tumorspheres, which are also characterized by elevated levels of stem cell markers [41]. It has been posited that the upregulation of *SERPINB9* might be contingent upon the activation of the MAPK signaling pathway. Corroborating this, our findings demonstrated that *SERPINB9* knockdown impeded the ERK1/2 cascade. Furthermore, we also observed downregulation of WNT signaling pathway following *SERPINB9* knockdown. These findings pinpointed the critical downstream signaling pathways regulated by *SERPINB9* and suggested that MAPK or WNT signaling inhibitors could be promising therapeutic strategies for non-seminoma patients with high *SERPINB9* expression. Moreover, *SERPINB9* is known to curtail the activity of granzyme B, a factor linked with CD8 T cell cytotoxicity. Consequently, *SERPINB9* might suppress immune responses, facilitating the evasion of cancer cells from immunosurveillance [42]. In alignment with this, our data also highlight a connection between *SERPINB9* and the downregulation of immunity. Given the pronounced pro-tumorigenic attributes of *SERPINB9* in EC, it emerges as a compelling candidate for therapeutic targeting in non-seminoma interventions.

While the presence of TLS has been established in a vast majority of solid tumors, the specific role of TLS within non-seminomas remains largely unexplored [21]. Divergent observations have been noted in prior literature concerning the relationship between TLS formation and tumor prognosis. Our findings underscore the utility of the TLS-associated gene signature as a robust prognostic indicator. The observed correlation between TLS formation and enhanced prognosis postulates the pivotal role of TLS in modulating tumor progression and its metastatic potential. Notably, we discovered that the expression of *SERPINB9* in EC contributed to the suppression of TLS, thereby fostering a tumor-promoting microenvironment. We also identified key chemokines including *IL6* and *CXCL13*, that hold promise for therapeutic strategies aimed at inducing TLS formation and amplifying antitumor immune responses in non-seminomas.

Our study is the first to profile the molecular signature of non-seminomas at single-cell resolution, offering an unparalleled insight into the transcriptional heterogeneity and immune microenvironment of these tumors. This understanding has paved the way for the identification of novel therapeutic targets.

## Methods and materials

### Collection of patient sample

Human primary non-seminoma samples were obtained from the Fudan University Shanghai Cancer Center. All patients were diagnosed in the pathology department with histopathology. This study was approved by the Ethics Committee of Shanghai Ninth People’s Hospital affiliated with Shanghai Jiao Tong University School of Medicine and the Research Ethics Committee of Shanghai Cancer Center, Fudan University. All participants have provided written informed consent.

### Tissue dissociation and single-cell suspension preparation

Tissues were preserved in the sCelLive^TM^ Tissue Preservation Solution (Singleron Bio Com, Nanjing, China) on ice within 30 min after resection. After wash with Hanks Balanced Salt Solution (HBSS), the specimens were digested with 2 ml sCelLive^TM^ Tissue Dissociation Solution (Singleron) using Singleron PythoN™ Automated Tissue Dissociation System (Singleron) at 37 °C for 15 min. The cell suspension was incubated with the GEXSCOPE^®^ red blood cell lysis buffer (Singleron) at 25 °C for 10 mins. The cells were centrifuged at 500 *g* for 5 min, followed by suspension with PBS. Finally, the cellular viability was tested with trypan blue (Sigma, United States).

### Single cell library preparation

The concentration of single-cell suspension was adjusted to 1 × 10^5^ cells/ml. The cell suspension was then loaded onto microfluidic devices with the Singleron Matrix^®^ Single Cell Processing System (Singleron). The GEXSCOPE^®^ Single Cell RNA Library Kits (Singleron) was used to construct the scRNA-seq libraries according to the protocol. Individual libraries were diluted to 4 nM and pooled. The pools were sequenced on Illumina Novaseq 6000 instrument with 150 paired end reads.

### Raw data processing, quality control and analysis

The CeleScope (v1.1.7) pipeline was applied to generate the filtered gene-cell matrix from the raw fastq files. In brief, Celescope was used to remove low quality reads. Cutadapt (v1.17) was used to trim poly-A tails and adaptor sequences. STAR (v2.6.1a) was used to align the reads to the reference genome GRCh38. Finally, the expression matrix was generated with featureCounts (v2.0.1). The crucial quality control metrics for single-cell experiments were provided in Table S1. The gene expression matrix was loaded into R with Seurat (v4.0.1). Genes that were expressed in three or fewer cells were excluded. The gene count per cell, UMI count per cell and percentage of mitochondrial transcripts was calculated. Seurat was used to remove low-quality cells with more than 20% of mitochondrial genes. Cells that expressed fewer than 200 genes or more than 6000 genes were also removed. Gene expression levels were normalized and scaled using *NormalizeData* and *ScaleData* functions. Top 2000 variable genes were selected with *FindVariableFeatures* function. *RunFastMNN* function from the R package SeuratWrappers (v0.3.0) was used to remove batch effects during the integration of samples. Using the top 30 principal components, cells were clustered using *FindClusters* function from Seurat. The function *runUMAP* from Seurat was used to perform UMAP dimensional reduction analysis.

### Open scRNA-seq datasets

The processed datasets of single naïve and primed human embryonic stem cells (ESC) were downloaded from the EBI database. The accession number is E-MTAB-6819 [43].

### InferCNV analysis

InferCNV (v1.6.0) was used to calculate chromosomal CNV score. Raw gene expression data extracted from the Seurat object, annotation information and gene/chromosome position file were prepared for the analysis. T/NK cells were selected as reference normal cells. The following parameters were used for the analysis: *cutoff* = *0.1, denoise* = *T*.

### Developmental trajectory

The Monocle 2 (v2.18.0) was used to build the pseudotime trajectory on the non-seminoma tumor cells. Top 2000 variable genes calculated by the *FindVariableFeatures* function from the Seurat package were used to order cells using the *setOrderingFilter* function. Dimensional reduction was performed using the *reduceDimension* function using the method “DDRTree”.

Integrated Seurat object of EC cells and ESCs were converted into CellDataSet object using the function *as.cell_data_set* from the R package SeuratWrapper. The functions *learn_graph* and *order_cells* were used to order cells in pseudotime. The root was set to naïve ESCs.

RNA velocity analysis was performed with the package velocyto (v0.17.17). Spliced and unspliced gene expression matrices were generated from bam files. The RNA velocity was estimated with the *RunVelocity* function with the default parameters: *deltaT* = *1, kCells* = *25, fit.quantile* = *0.02*. Then, the velocity was visualized on the UMAP embedding of the Seurat object of non-seminoma tumor cells.

### Single-cell regulatory network inference and clustering (SCENIC)

The pySCENIC (v0.12.0) package was applied on normalized gene expression matrix of the non-seminoma tumor cells. Gene regulatory network was constructed with Arboreto (v0.1.6) using the GRNboost2 method. The cisTarget human motif database, motifs-v9-nr.hgnc-m0.001-o0.0.tbl, was used in the *pyscenic ctx* function with default parameters. The *auc* argument was further used to score the single-cell activity of gene signatures. The activity scores were further added to Seurat object with the *CreateAssayObject* function.

### Differential gene expression analysis

The FindMarkers function using the Wilcox likelihood-ratio test was used to calculated the differentially expressed genes (DEG) between cell clusters. Genes with average log2 fold change > 0.25 and expressed in more than 10% of the cells were selected as markers of cell clusters.

### Pathway enrichment analysis

Functional enrichment analysis was performed using the Metascape website with default settings (https://metascape.org/gp/index.html#/main/step1). Gene set enrichment analysis (GSEA) was performed using the R package clusterProfiler (v3.18.1). Gene sets were obtained from the Molecular Signature Database (MSigDB).

### Gene module detection

Hotspot (v1.1.1) was used to identify highly correlated gene modules. The expression matrix of all of the non-seminoma tumor cells (including EC and teratoma) was used as the input. The K-nearest-neigh is computed using the *create_knn_graph* function using the following parameter: *n_neighbors* = *30*. Informative genes with FDR < 0.05 were kept for further analysis. Genes were grouped into modules with the function create_modules using the following parameters: *min_gene_threshold* = *15 and fdr_threshold* = *0.05*.

### TCGA and survival analysis

The FPKM data and clinical metadata of TCGA TGCT datasets were downloaded using the R package TCGAbiolinks. Non-seminoma samples were selected according to annotation information for further survival analysis. Tumor samples were scored using the singscore (v1.10.0) package with specific gene sets. Based on the scores, the patients were classified into high-risk and low-risk groups using the function *surv_cutpoint* from the package survminer (v0.4.9). The function *survfit* from the package survival (v3.2-7) was used to fit survival curves with Kaplan-Meier method. The function *surv_pvalue* was used to compared the survival curves using the Wilcoxon method. The function *ggsurvplot* was further used for visualization.

### Cell-cell interaction analysis

We used CellChat (v1.1.3) with default arguments to reveal the interaction between cell clusters. The analysis was performed separately in non-metastatic and metastatic datasets.

### Total RNA extraction, RNA library construction and sequencing

Total RNA was extracted according to the manual of RNAiso Plus (TaKaRa, Japan). RNA was quantified using the Qubit 4.0 instrument (Invitrogen, USA). The integrity of RNA was tested with electrophoresis.

The libraries were constructed using VAHTS Universal V6 RNA-seq Library Prep Kit per manufacturer’s instructions (Vazyme, China). Libraries were sequenced on the NovaSeq 6000 (Illumina, USA).

### Real-time PCR

After extraction of total RNA from cell lines using RNAiso Plus (TaKaRa, Japan), cDNA was transcribed reversely using the PrimeScript^®^ RT Reagent Kit with gDNA Eraser (TaKaRa, Japan). The target sequences were amplified with real-time PCR using TB Green Premix Ex Taq II in Roche Light Cycler 480 Real-Time PCR detector. The primers used to quantify gene expression levels in this study were listed in Table S2. Comparative threshold cycle (2^−ΔΔCT^) method was used to calculate the relative mRNA level with *RPS29* as the reference gene.

### Bulk RNA-seq analysis

The quality of raw fastq files was inspected using fastqc (v0.11.9). The adapters and low-quality reads were trimmed with the software trimmomatic (v0.39). The reads from RNA-seq were aligned to the human reference genome (GRCh38.p13) with the software STAR (v020201). Subsequently, the mapped reads were quantified as counts using featureCounts (v2.0.1). The R package edgeR (v3.32.1) was used to identify the differentially expressed genes.

### Cell culture

The human EC cell line NCCIT was obtained from ATCC (CRL-2073). The human EC cell line NTERA-2 was obtained from Immocell (Xiamen, China). NCCIT was cultured in RPMI-1640 medium supplemented with 10% fetal bovine serum (FBS). NTERA-2 was cultured in DMEM (high glucose) supplemented with 10% FBS. The cells were cultured in an incubator with 5% CO_2_ at 37 °C in a humidified atmosphere.

### Stable cell line construction

Human *SERPINB9* shRNAs were designed using WI siRNA selection program (http://sirna.wi.mit.edu). The scramble sequences were generated by randomly disrupting the sh*SERPINB9* sequences to serve as negative controls. Both sh*SERPINB9* and scramble sequences are listed in Table S2. These sequences were cloned into pGreenPuro vector. The viral supernatant was harvest and used to transduce the target cells. The concentration of the puromycin was 3 µg/ml for the selection of stable cell line.

### Transwell migration assay

The 24-well chamber with 8 µm pore polycarbonate membrane insert was used for the Transwell migration assay. The bottom chambers were filled with 600 µl medium supplemented with 10% FBS. In the upper chamber, a total of 5 × 10^4^ cells were seeded in 100 µl of FBS-free cell culture medium. The chamber was incubated in the incubator with 5% of CO2 at 37 °C for 24 h. Subsequently, cells were fixed with 4% paraformaldehyde. PBS was used to wash the cells three times. Then the cells were stained with 0.05% crystal violet for 15 mins. Cells on the upper side of the membrane were removed by cotton swab. A microscope was used to capture the picture. ImageJ (v1.53) was used to analyze the area of cells that penetrated the membrane.

### Drug sensitivity assay

Cisplatin was purchased from Sigma. The CellTiter-Lumi Luminescent Cell Viability Assay Kit (Beyotime, China) was used to assess the drug sensitivity of cell lines to cisplatin. Cells were seeded at a density of 3000 cells per well in 96-well plates, incubated overnight, and then exposed to serial dilutions of cisplatin for 72 h. After incubation, 100 µl of the detection reagent was added to each well. The plates were vortexed for 2 min and incubated at room temperature for 10 min. Chemiluminescence was measured using the VICTOR NIVO plate reader (PerkinElmer, USA). Nonlinear regression analysis was used to calculate IC50 values using GraphPad Prism software.

### Cell proliferation assay

The Incucyte Live-Cell Analysis System (Sartorius, USA) was used to kinetically monitor cell growth according to the manufacturer’s instructions. In brief, NCCIT (3000/well) and NTERA-2 (3000/well) were seeded in 96-well plates and monitored over 160 h. Growth curves were generated from data points collected at 4 h intervals during imaging.

### Embryoid body formation assay

The embryoid body formation assay was performed according to previous publication [44]. A total of 5 × 10^5^ NCCIT cells were plated in a 60 mm ultra-low attachment plate (Costar, USA) and cultured in Opti-MEM for 2 days to induce the formation of embryoid body. Embryoid bodies were embedded in 2% agarose. Then, the embedded embryoid bodies were dehydrated and further embedded in paraffin. The sections from the paraffin embedded embryoid bodies were used for further IF.

### Cell line-derived xenograft (CDX) tumor model

All animal experimental procedures were conducted in accordance with protocols approved by the Animal Care and Use Committee of Shanghai Ninth People’s Hospital, Shanghai Jiao Tong University School of Medicine (SH9H-2019-A727-1). The procedures complied with the Animals (Scientific Procedures) Act, 1986 (UK) (amended 2013). The mice were housed in individually ventilated cages (five per cage) under specific pathogen-free (SPF) conditions at Ninth People’s Hospital animal facility. They were maintained in a temperature-controlled environment with a 12 h light/dark cycle and given ad libitum access to food and water. Animal weights were recorded every other day.

Six-week-old NOD-SCID mice were obtained from the Experimental Animal Centre of Shanghai Institutes of Biological Sciences (Shanghai, China). Mice were injected subcutaneously with NCCIT cells (1 × 10^7^ cells in 100 µl PBS), transfected with either *SERPINB9*-shRNA or a control vector, mixed with Matrigel (1:1, Corning, USA), into their flanks. Tumor growth was monitored periodically using a digital caliper, with the volume calculated by the formula: (length x width^2^)/2. At the study endpoints, the mice were euthanized, and tumor tissues were excised, weighed, and photographed. The tissues were then fixed in paraformaldehyde (PFA) and embedded in paraffin for subsequent assays.

### Limiting dilution assay

Four-week-old NOD-SCID mice were obtained from the Experimental Animal Centre of Shanghai Institutes of Biological Sciences (Shanghai, China). Mice were injected subcutaneously with 5 × 10^4^, 5 × 10^3^ or 5 × 10^2^ NCCIT cells, which were transfected with either *SERPINB9*-shRNA or a control vector. The cells were mixed with Matrigel (1:1, Corning, USA) and injected into the left or right flanks of NOD-SCID mice. The mice were sacrificed after 5-6 weeks. The frequency of cancer stem cells (CSCs) was then calculated using Extreme Limiting Dilution Analysis (ELDA) (https://bioinf.wehi.edu.au/software/elda/).

### Hematoxylin and eosin (H&E) staining

Paraffin sections were baked at 60 °C for 1 h before deparaffinizing in xylene for 20 min twice. Sections were rehydrated through soaking in 100% ethanol for 2 min twice, then in 95%, 70% and 50% ethanol, each for 2 min. After rinsing in tap water and distilled water, Hematoxylin (E607318-0200A, BBI, China) was used for staining for 10 min. Sections were immersed in 1% hydrochloric acid in alcohol for 3 s for differentiation. For bluing, the sections were rinsed for 10 min. After immersed in 95% ethanol for 10 s, eosin (E607318-0200B, BBI, China) was used for staining for 3 s. After dehydration, sections were cleared in xylene three times, each for 5 min. The tissue sections were then mounted in neutral balsam and sealed with microscope cover slips. The tissues were then observed using an optical microscope (SLIDEVIEW VS200, Olympus, Japan).

### Immunohistochemistry

Formalin-fixed paraffin-embedded (FFPE) sections were collected. IHC staining was used to evaluated the protein expression levels. Primary antibodies used in this study are listed in Table S3. H-score was calculated by multiplying the percentage of stained cells and staining intensity according to previous publication [45]. The intensity scores included 0 (no evidence of staining), 1 (weak staining), 2 (moderate staining), 3 (strong staining). The F-IHC conducted in CDX tumor tissues was according to the Opal 4-Color Manual IHC Kit following the manufacturer’s protocol (PerkinElmer, USA).

### Enzyme-linked immunosorbent assay (ELISA)

Subsequent to the collection of supernatants from the cell culture, the protein concentrations of the cytokines IL6, IL11, IL15, CCL2, CCL5, and CXCL13 were quantified. This quantification was performed using human-specific ELISA kits (Proteintech, USA). The optical density (OD) values were measured at a wavelength of 450 nm utilizing the VICTOR NIVO plate reader (PerkinElmer, USA) to determine the concentrations of the cytokines.

### Statistical analysis

Statistical analyses were performed on R (4.0.1) and GraphPad Prism (v9.5.0). Shapiro-Wilk test was used to test the normality of data. For data following a normal distribution, Welch’s *t*-test was applied to determine statistical significance. Conversely, for data that did not conform to a normal distribution, the Wilcoxon rank-sum test was utilized. Normally distributed data are presented as the mean ± SD, while non-normally distributed data are presented as the median and IQR. All experiments were repeated at least three times. Prior experiments with comparable methodologies were used to determine sample sizes. *P* < 0.05 was considered statistically significant.

## Supplementary information

Supplementary legends

Supplementary figures

Table S1

Table S2

Table S3

## Data Availability

The raw sequence data of bulk RNA-seq and scRNA-seq reported in this paper have been deposited in the Genome Sequence Archive in National Genomics Data Center, China National Center for Bioinformation / Beijing Institute of Genomics, Chinese Academy of Sciences (GSA-Human: HRA005581/HRA005800) that are publicly accessible at https://ngdc.cncb.ac.cn/gsa-human.
