# Supplementary material for: Single-cell landscape identified SERPINB9 as a key player contributing to stemness and metastasis in non-seminomas
Source: Cell Death Dis. 2024 Nov 11;15(11):812. doi: 10.1038/s41419-024-07220-5 (PMC11555415; doi:10.1038/s41419-024-07220-5)
Supplement: Supplementary file 1 — Supplementary legends [file 41419_2024_7220_MOESM1_ESM.docx]

**Supplementary legends**

**Figure S1. Density plot showing the expression of canonical marker genes**

**Figure S2. Heatmap showing copy number variation in samples from P1-P3**

**Figure S3.** **HE and IHC staining showing classical markers including OCT4 and CD30 of EC for P2-4.**

**Figure S4. *SERPINB9* is associated stemness maintenance in EC**

**(a)** ATAC-seq datasets showing peaks on the promoter regions of *SERPINB9* in hESC but not in differentiated cells. **(b)** Boxplot illustrating the increased expression of *SERPINB9* in stage II-III patients compared to stage I patients based on the TCGA non-seminoma dataset (P value: Wilcoxon test). **(c)** Validation of the knockdown of *SERPINB9* in NCCIT by qPCR (P value: Welch’s t-test). **(d)** Validation of the knockdown of *SERPINB9* in NTERA-2 by qPCR (P value: Welch’s t-test). **(e)** Transwell migration assay showing decrease of migratory capability of NCCIT after knockdown of *SERPINB9* using two distinct shRNAs (scale bar: 50 µm; P value: Welch’s t-test). **(f)** Transwell migration assay showing decrease of migratory capability of NTERA-2 after knockdown of *SERPINB9* using two distinct shRNAs (scale bar: 50 µm; P value: Welch’s t-test). **(g)** Drug sensitivity assay showing decrease of IC50 after the knockdown of *SERPINB9* using two distinct shRNAs (IC50: 3.696 µM vs 2.322 µM (shCtrl-2 vs sh*SERPINB9*-2), 3.438 µM vs 2.180 µM (shCtrl-3 vs sh*SERPINB9*-3); P value: Welch’s t-test). **(h)** Drug sensitivity assay showing decrease of IC50 after the knockdown of NTERA-2 using two distinct shRNAs (IC50: 2.402 µM vs 2.040 µM (shCtrl-2 vs sh*SERPINB9*-2), 2.652 µM vs 1.925 µM (shCtrl-3 vs sh*SERPINB9*-3); P value: Welch’s t-test). **(i)** Cell growth curves of NCCIT cells following transfection with shCtrl and sh*SERPINB9* shRNAs (P value: Welch’s t-test). **(j)** Cell growth curves of NCCIT cells following transfection with shCtrl-2 and sh*SERPINB9*-2 shRNAs (P value: Welch’s t-test). **(k)** Cell growth curves of NCCIT cells following transfection with shCtrl-3 and sh*SERPINB9*-3 shRNAs (P value: Welch’s t-test). **(l)** Cell growth curves of NTERA-2 cells following transfection with shCtrl and sh*SERPINB9* shRNAs (P value: Welch’s t-test). **(m)** Cell growth curves of NTERA-2 cells following transfection with shCtrl-2 and sh*SERPINB9*-2 shRNAs (P value: Welch’s t-test). **(n)** Cell growth curves of NTERA-2 cells following transfection with shCtrl-3 and sh*SERPINB9*-3 shRNAs (P value: Welch’s t-test).

**Figure S5. *SERPINB9* is associated with ERK1/2 and WNT signaling pathways activation.**

**(a)** Bar plot showing decreased number of embryoid bodies after *SERPINB9* knockdown (P value: Welch’s t-test). **(b)** Bar plot showing decreased fluorescence intensity of SERPINB9 after *SERPINB9* knockdown (P value: Welch’s t-test). **(c)** Bar plot showing decreased fluorescence intensity of OCT4 after *SERPINB9* knockdown (P value: Welch’s t-test). **(d)** Bar plot showing decreased fluorescence intensity of AFP after *SERPINB9* knockdown (P value: Welch’s t-test). **(e)** Bar plot showing decreased fluorescence intensity of ACTA2 after *SERPINB9* knockdown (P value: Welch’s t-test). **(f)** Bar plot showing decreased fluorescence intensity of CD57 after *SERPINB9* knockdown (P value: Welch’s t-test). **(g)** Heatmap showing downregulation of genes associated with ERK1/2 signaling after *SERPINB9* knockdown in RNA-seq dataset. **(h)** Bar plot showing downregulation of genes associated with ERK1/2 signaling after *SERPINB9* knockdown in embryoid bodies validated by qPCR (P value: Welch’s t-test). **(i)** Heatmap showing downregulation of genes associated with WNT signaling after *SERPINB9* knockdown in RNA-seq dataset. **(j)** Bar plot showing downregulation of genes associated with WNT signaling after *SERPINB9* knockdown in embryoid bodies validated by qPCR (P value: Welch’s t-test). **(k)** GSEA analysis showing the enrichment of the ERK1/2 cascade in samples with higher *SERPINB9* expression from TCGA non-seminoma dataset. **(l)** GSEA analysis showing the enrichment of the canonical WNT signaling pathway in samples with higher *SERPINB9* expression from TCGA non-seminoma dataset. **(m)** IF analysis showing reduced p-ERK expression in embryoid bodies following *SERPINB9* knockdown (scale bar: 50 µm; P value: Welch’s t-test). **(n)** IF analysis showing reduced β-Catenin expression in embryoid bodies following *SERPINB9* knockdown (scale bar: 50 µm; P value: Welch’s t-test).

**Figure S6. *SERPINB9* is associated with stemness maintenance in vivo.**

**(a)** HE staining of tumors from the limiting dilution assay (scale bar: 50 µm). **(b)** F-IHC analysis showing successful *SERPINB9* knockdown (scale bar: 50 µm; P value: Welch’s t-test). **(c)** F-IHC analysis showing reduced OCT4 expression after *SERPINB9* knockdown (scale bar: 50 µm; P value: Welch’s t-test). **(d)** Representative images of tumors from the CDX models. Bar plot showing the weight of individual tumor from each group (P value: Welch’s t-test). **(e)** Representative images of HE staining of the CDX tumor tissues (scale bar: 50 µm). **(f)** Bar plot showing decreased fluorescence intensity of SERPINB9 after *SERPINB9* knockdown (P value: Welch’s t-test). **(g)** Bar plot showing decreased fluorescence intensity of OCT4 after *SERPINB9* knockdown (P value: Welch’s t-test). **(h)** Bar plot showing decreased fluorescence intensity of AFP after *SERPINB9* knockdown (P value: Welch’s t-test). **(i)** Bar plot showing decreased fluorescence intensity of ACTA2 after *SERPINB9* knockdown (P value: Welch’s t-test). **(j)** Bar plot showing decreased fluorescence intensity of CD57 after *SERPINB9* knockdown (P value: Welch’s t-test). **(k)** F-IHC analysis showing reduced p-ERK expression after *SERPINB9* knockdown (scale bar: 50 µm; P value: Welch’s t-test). **(l)** F-IHC analysis showing reduced β-Catenin expression after *SERPINB9* knockdown (scale bar: 50 µm; P value: Welch’s t-test).

**Figure S7. Marker genes of immune and mesenchymal cells.**

**(a)** UMAP plot showing expression of marker genes associated with mesenchymal cell subsets. **(b)** UMAP plot showing expression of Tph-associated markers in T/NK cells. **(c)** UMAP plot showing expression of marker genes associated with myeloid cell subsets.

**Figure S8. Cellular interaction in non-seminomas**

**(a)** Scatter plot showing inverse correlation between samples scored by gene signatures derived from *IGF2*+ myofibroblasts and B cells in TCGA TGCT non-seminoma dataset using Spearman’s correlation (P value and correlation coefficient: Spearman’s correlation test). **(b)** Expression of CD3, CD20 and CD21 in metastatic sample (scale bar: 100 µm). **(c)** Cell-cell communication among cell clusters in metastatic non-seminomas.

**Table S1. Crucial quality control metrics for scRNA-seq experiments**

**Table S2. Primers used in this study**

**Table S3. Antibodies used in this study**
