# Supplementary figures and images for: Single-cell landscape identified SERPINB9 as a key player contributing to stemness and metastasis in non-seminomas
Source: Cell Death Dis. 2024 Nov 11;15(11):812. doi: 10.1038/s41419-024-07220-5 (PMC11555415; doi:10.1038/s41419-024-07220-5)

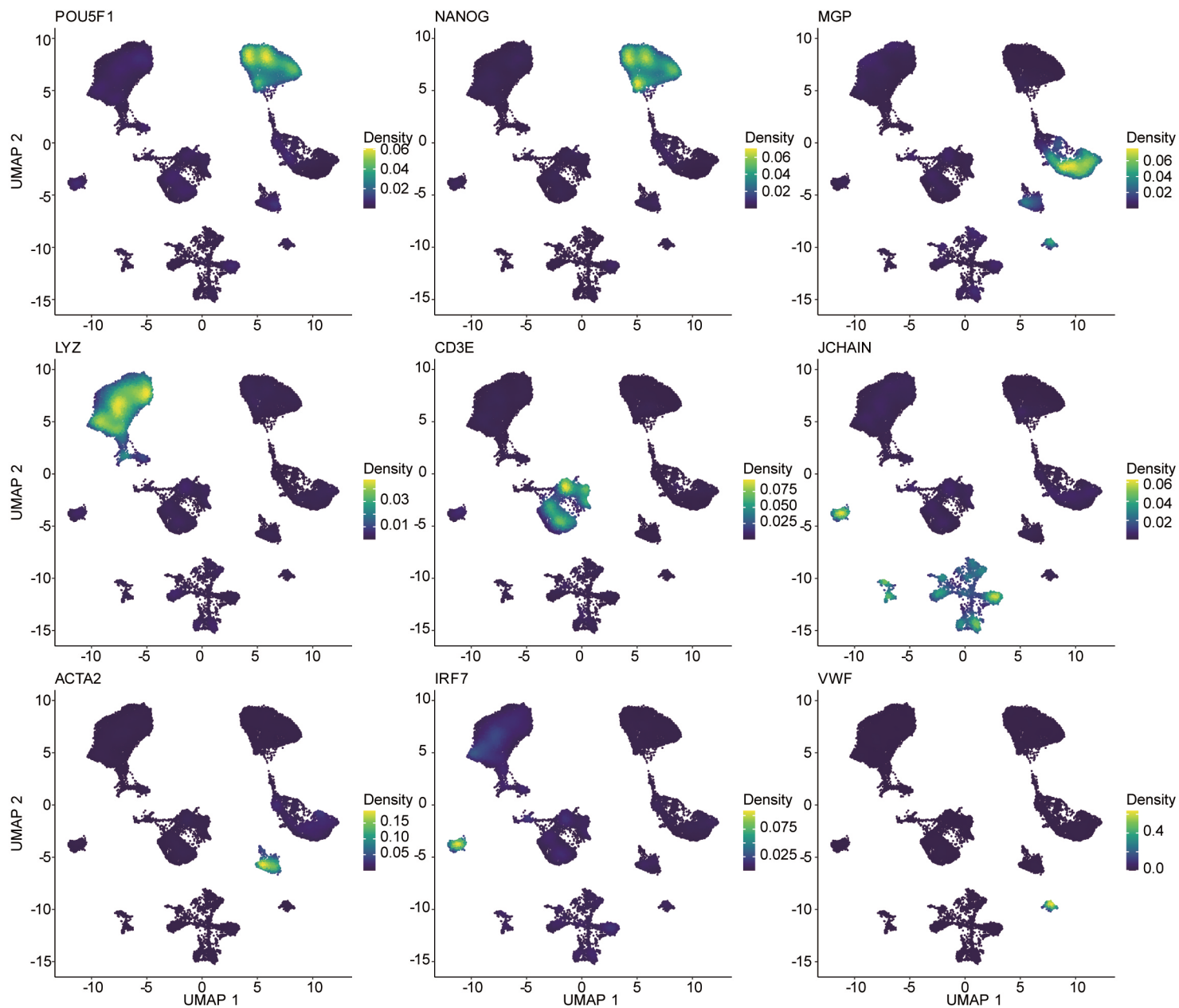

P1

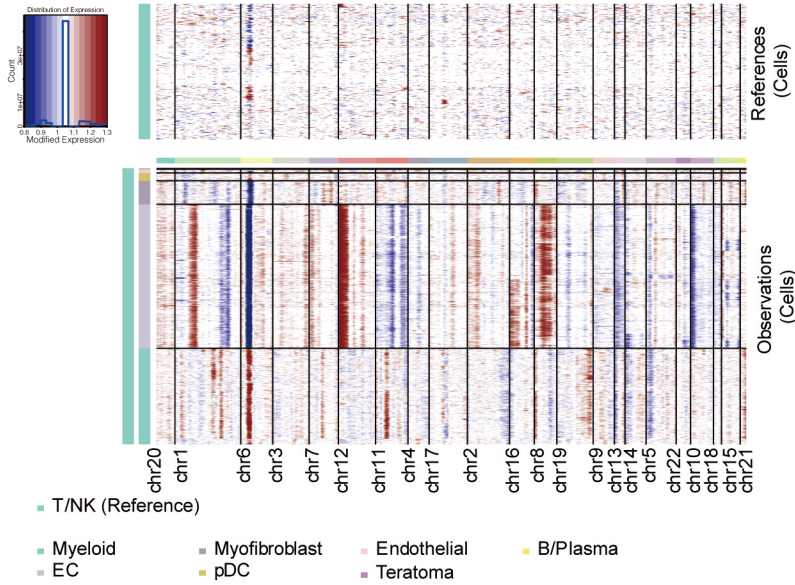

P2

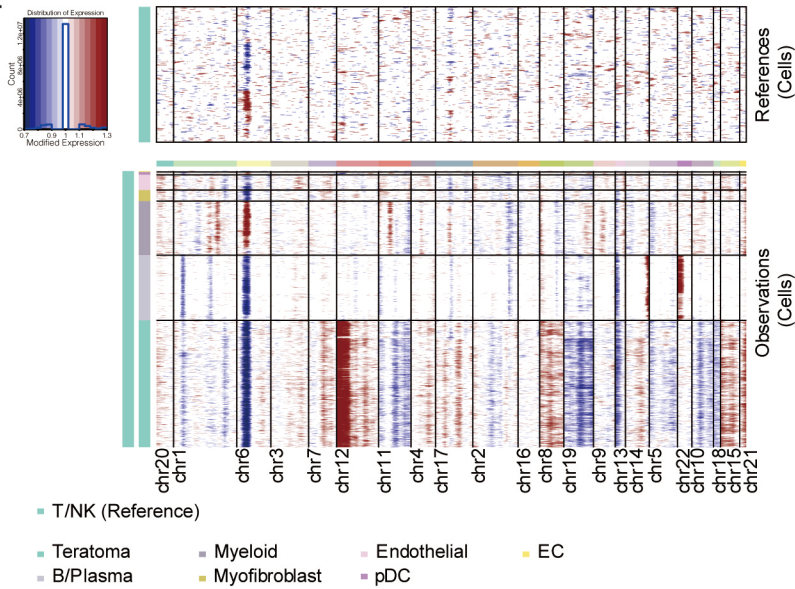

P3

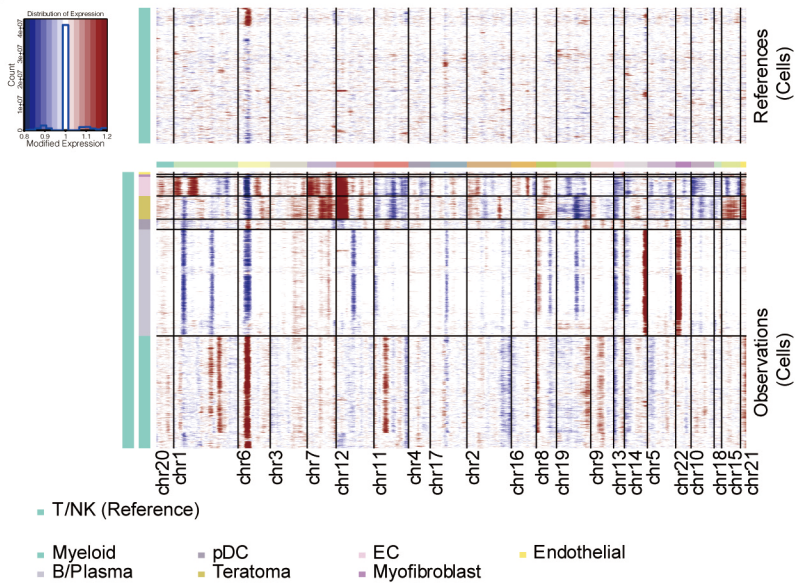

P2

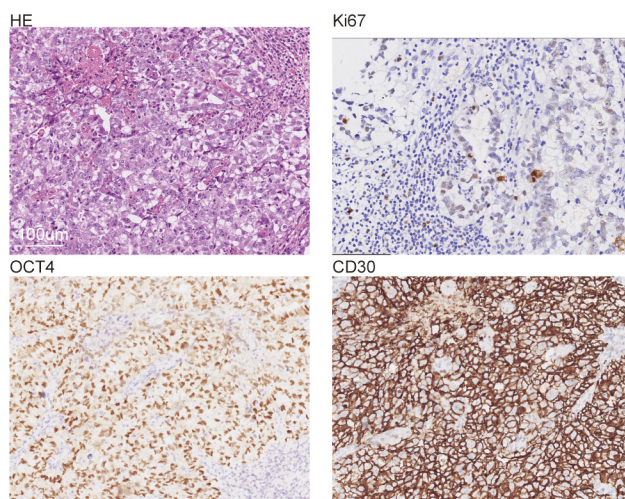

P3

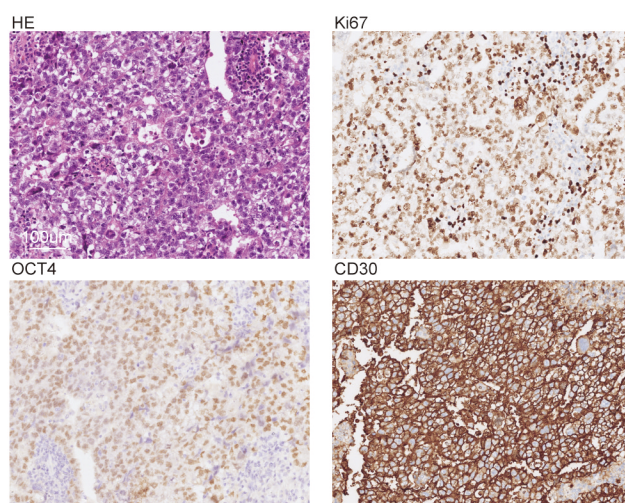

P4

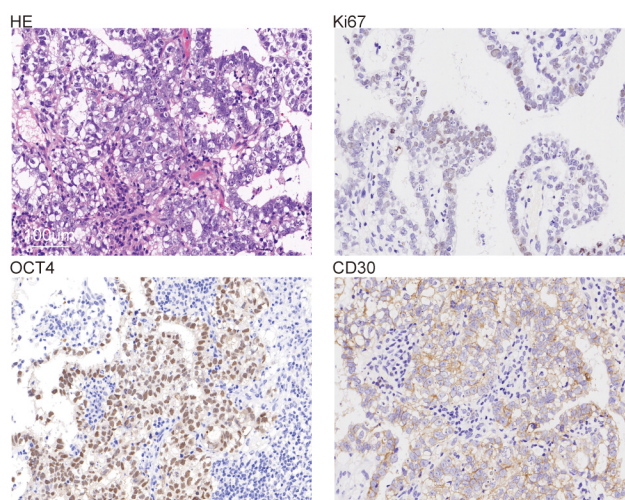

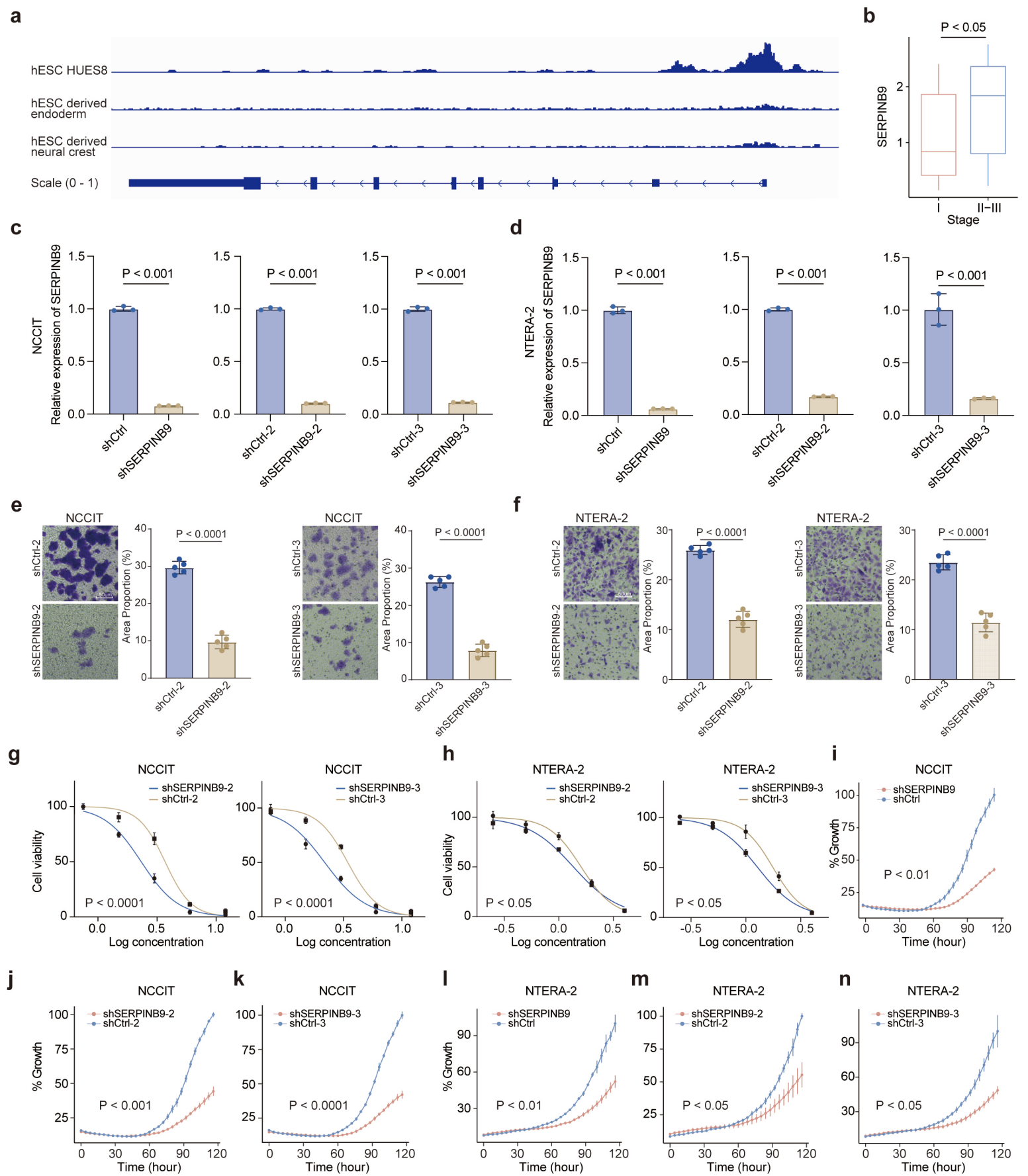

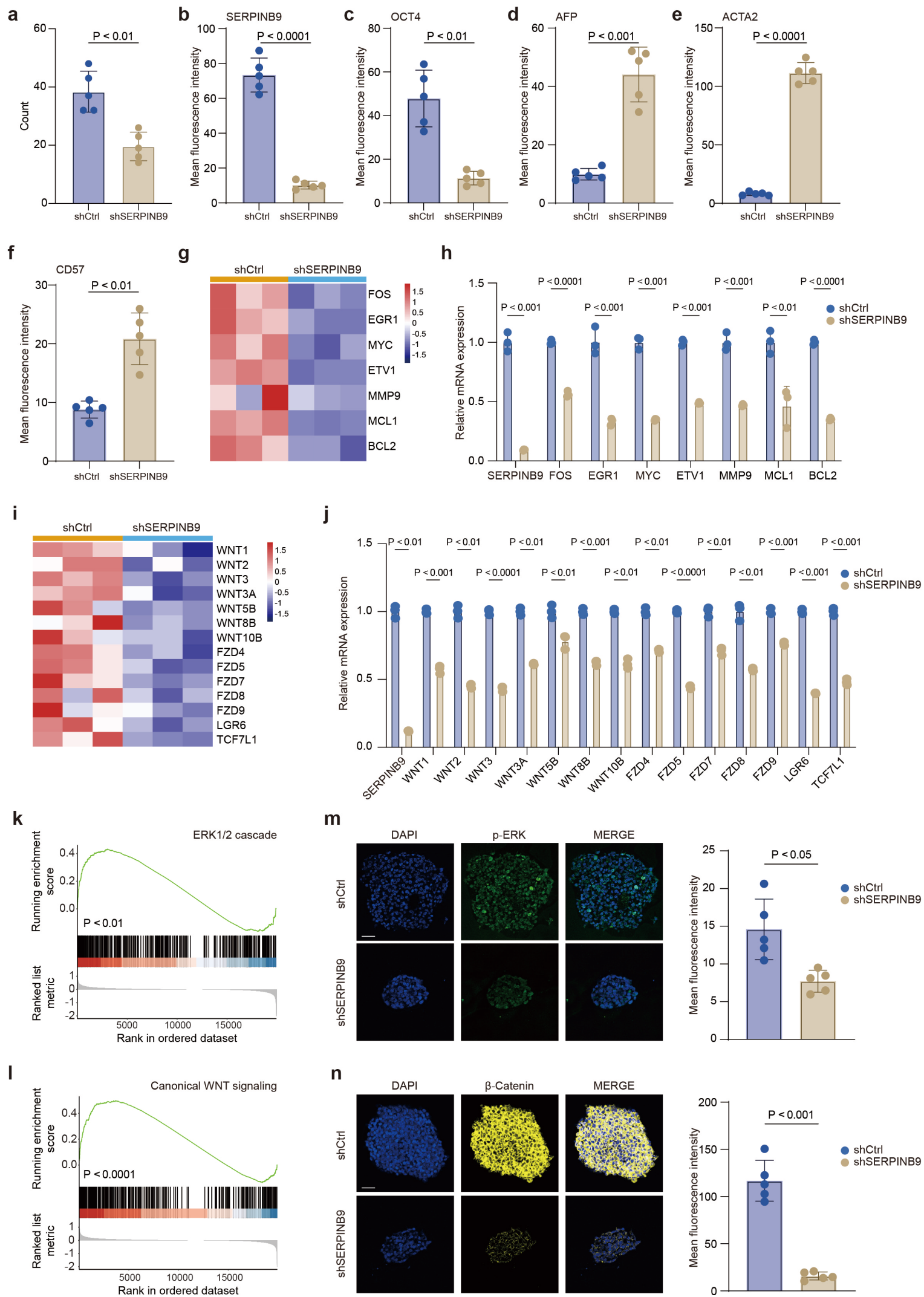

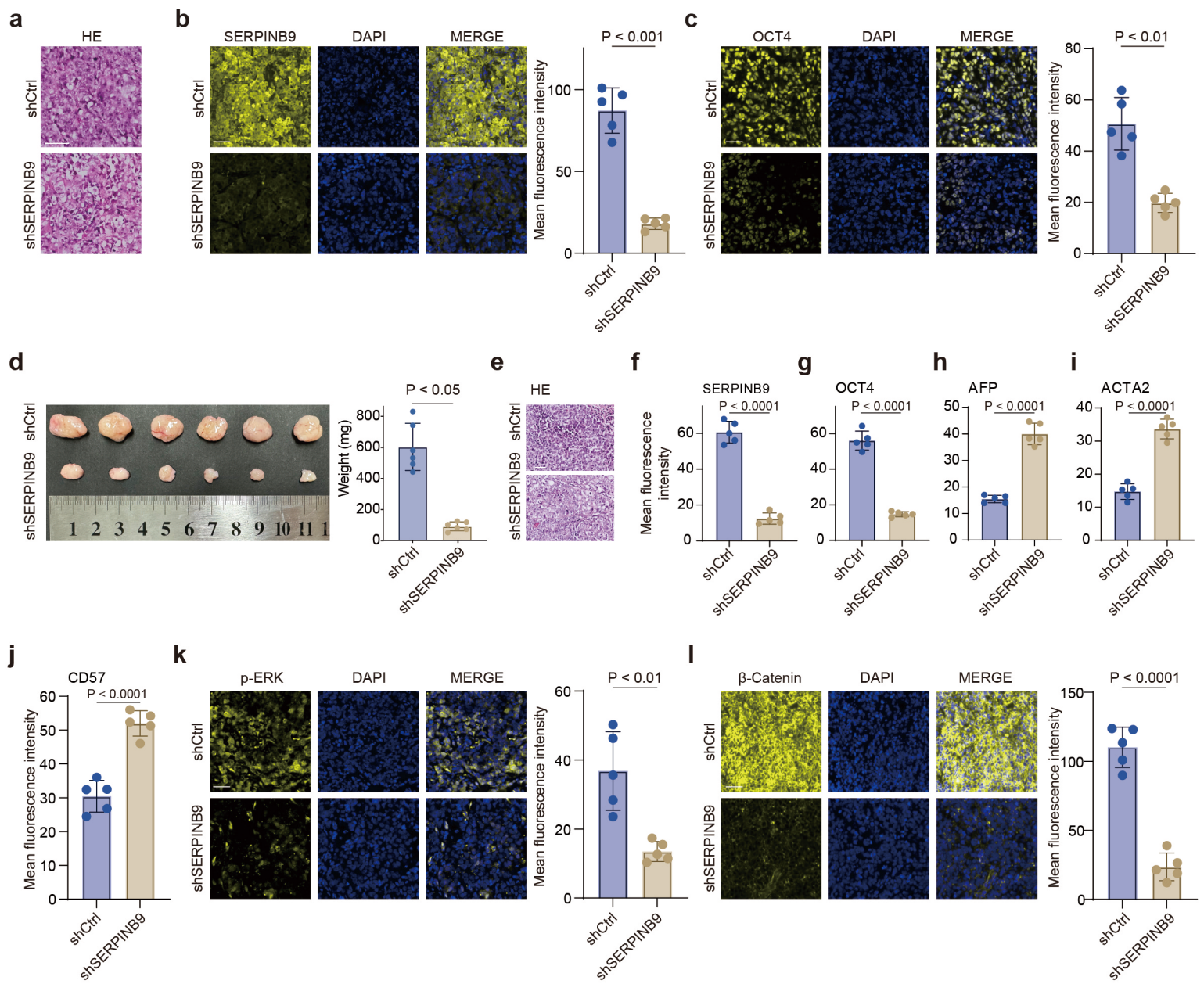

**a**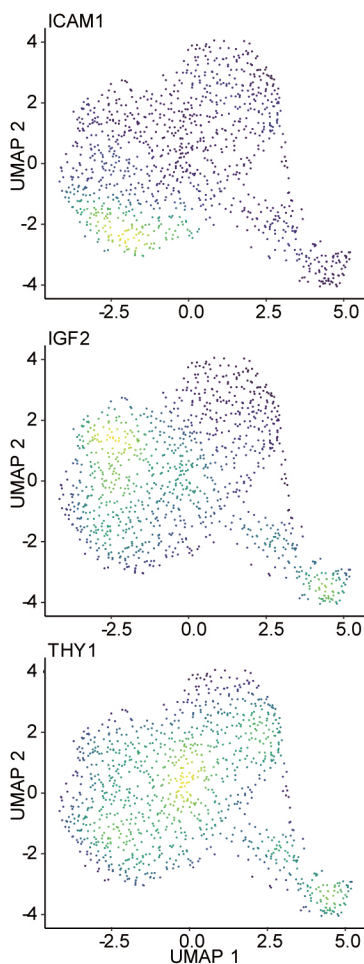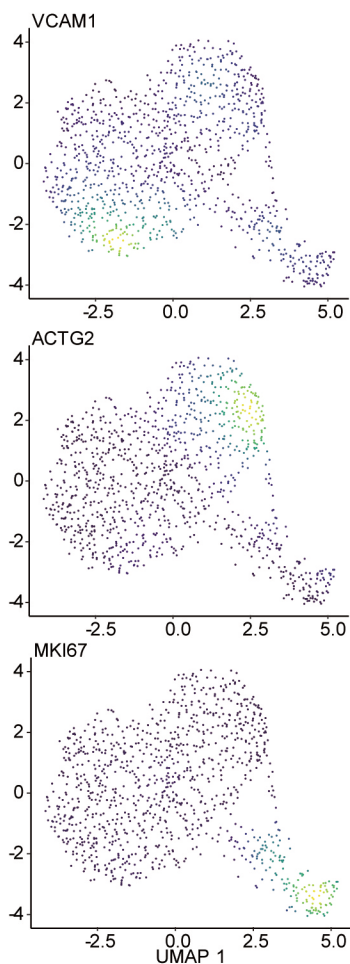**b**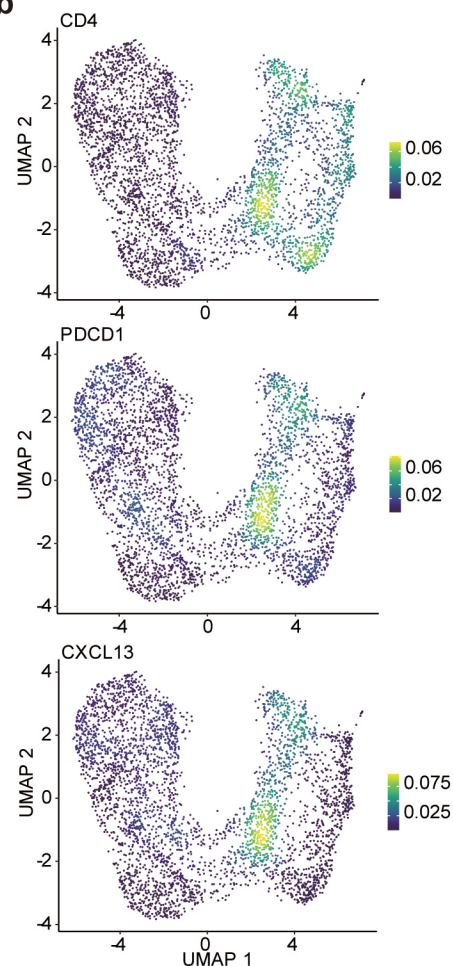**c**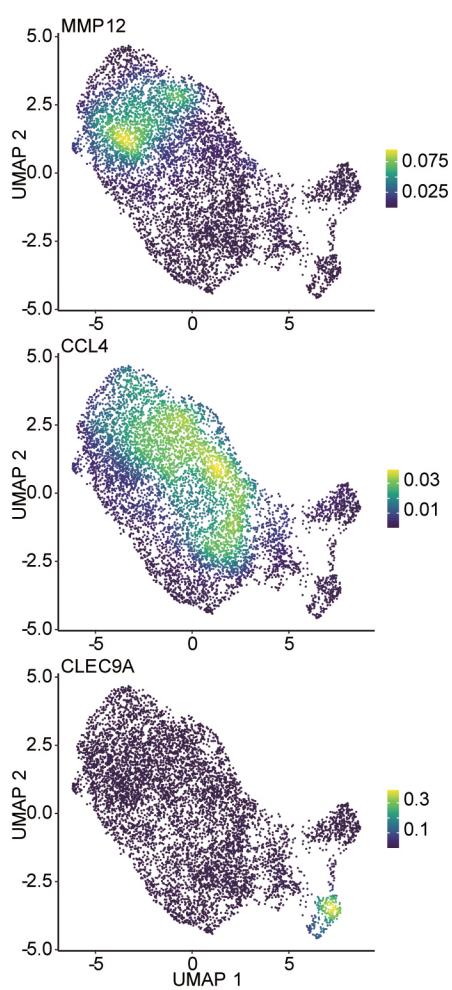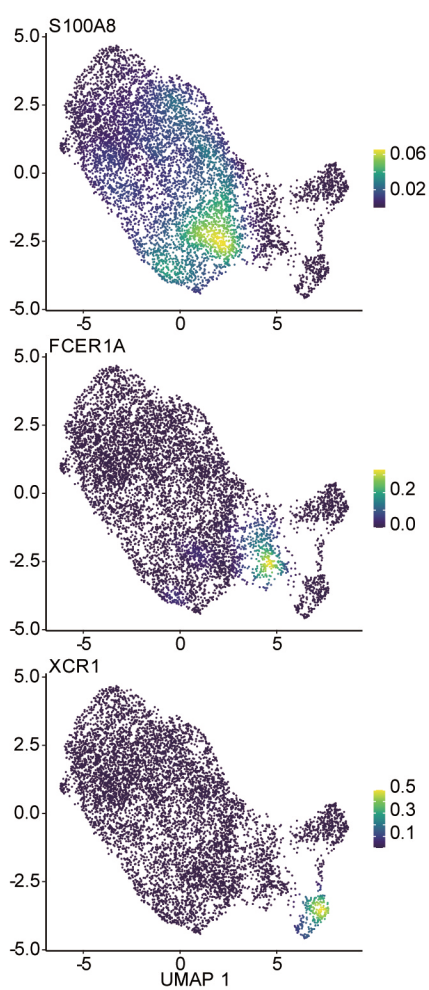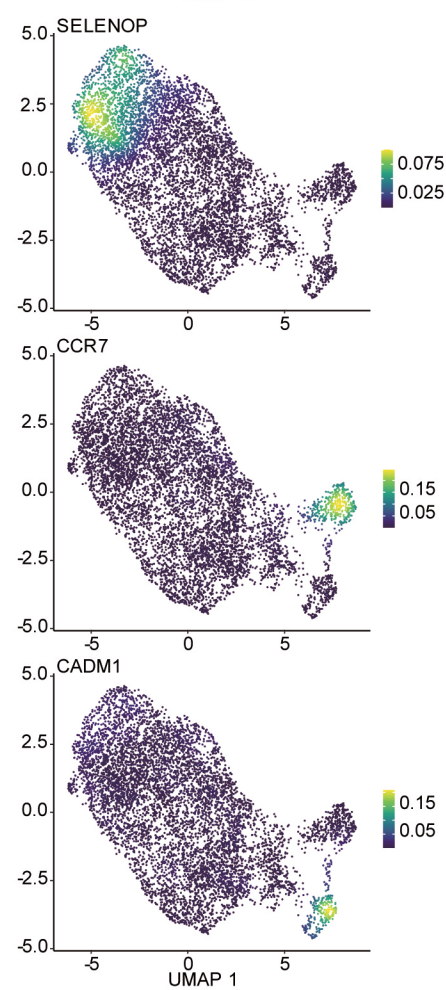

**a**

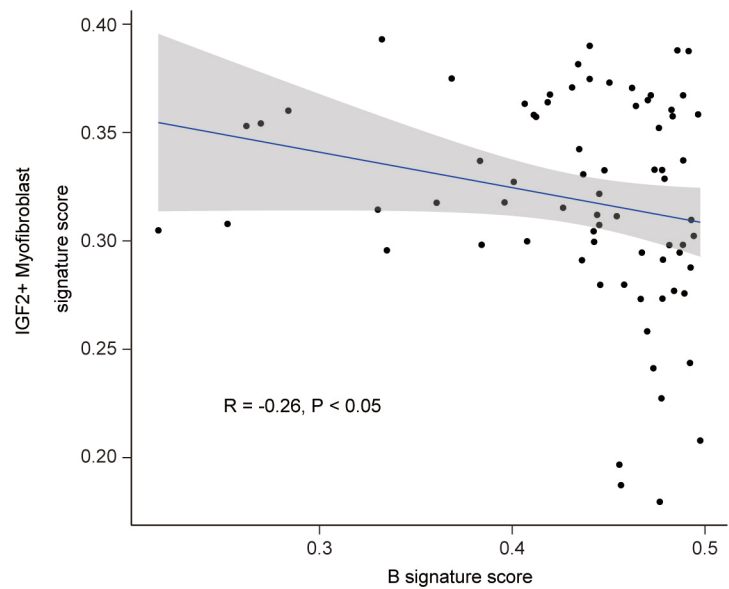

**b**

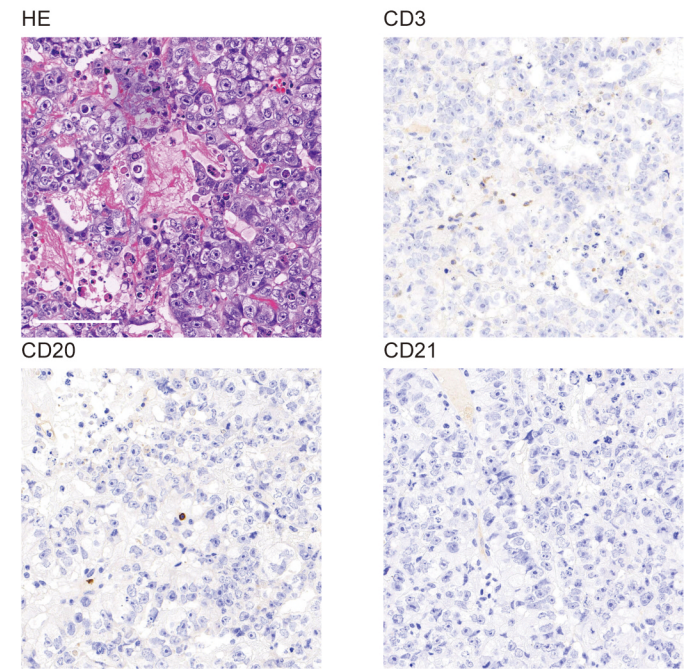

**c**

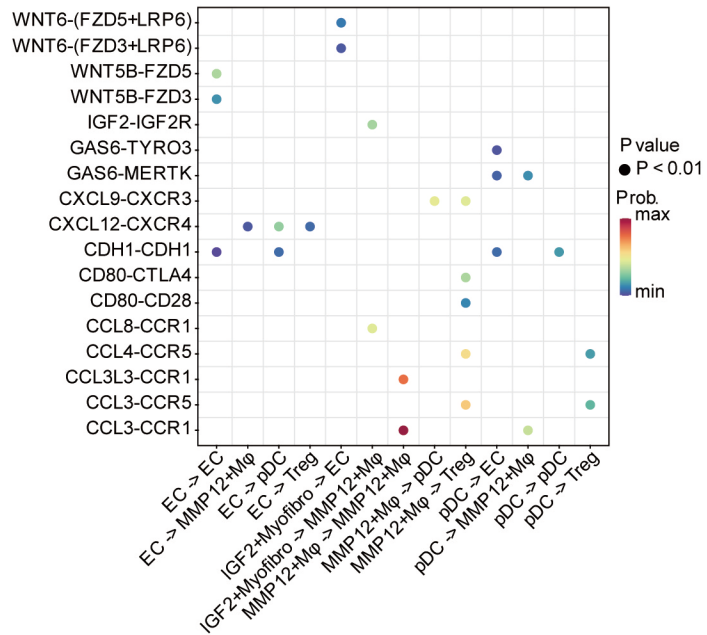

Supplement: Supplementary file 2 — Supplementary figures [file 41419_2024_7220_MOESM2_ESM.pdf]
